# Clinical Outcomes of a Randomized Trial of Adaptive Plan-of-the-Day Treatment in Patients Receiving Ultra-hypofractionated Weekly Radiation Therapy for Bladder Cancer

**DOI:** 10.1016/j.ijrobp.2020.11.068

**Published:** 2021-06-01

**Authors:** Robert Huddart, Shaista Hafeez, Rebecca Lewis, Helen McNair, Isabelle Syndikus, Ann Henry, John Staffurth, Monisha Dewan, Catalina Vassallo-Bonner, Syed Ali Moinuddin, Alison Birtle, Gail Horan, Yvonne Rimmer, Ramachandran Venkitaraman, Vincent Khoo, Anita Mitra, Simon Hughes, Stephanie Gibbs, Gaurav Kapur, Angela Baker, Vibeke Nordmark Hansen, Emma Patel, Emma Hall, HYBRID Investigators

**Affiliations:** ∗ The Institute of Cancer Research, London, United Kingdom; † Royal Marsden NHS Foundation Trust, London, United Kingdom; ‡ Radiotherapy Department, Oxford University Hospitals NHS Foundation Trust, Oxford, United Kingdom; § Leeds Teaching Hospitals NHS Trust, Leeds, United Kingdom; ‖ Velindre University NHS Trust, Cardiff, United Kingdom; ¶ Patient representative, Institute of Cancer Research, London, United Kingdom; # Academic unit of Oncology, Department of Oncology and Metabolism, Medical School, University of Sheffield, Sheffield, United Kingdom; ∗∗ Lancashire Teaching Hospitals NHS Foundation Trust, Preston, United Kingdom; †† Queen Elizabeth Hospital Kings Lynn NHS Trust, Kings Lynn, United Kingdom; ‡‡ Cambridge University Hospitals NHS Foundation Trust, Cambridge, United Kingdom; §§ East Suffolk and North Essex NHS Foundation Trust, Ipswich, United Kingdom; ‖‖ University College London Hospitals NHS Foundation Trust, London, United Kingdom; ¶¶ Guy's and St Thomas' NHS Foundation Trust, London, United Kingdom; ## Barking, Havering and Redbridge University Hospitals NHS Trust, Romford, United Kingdom; ∗∗∗ Norfolk and Norwich University Hospitals NHS Foundation Trust, Norwich, United Kingdom; ††† Odense University Hospital, Odense, Denmark; ‡‡‡ Radiotherapy Trials Quality Assurance Group, Mount Vernon Cancer Centre, Northwood, United Kingdom

## Abstract

**Purpose:**

Hypofractionated radiation therapy can be used to treat patients with muscle-invasive bladder cancer unable to have radical therapy. Toxicity is a key concern, but adaptive plan-of the day (POD) image-guided radiation therapy delivery could improve outcomes by minimizing the volume of normal tissue irradiated. The HYBRID trial assessed the multicenter implementation, safety, and efficacy of this strategy.

**Methods:**

HYBRID is a Phase II randomized trial that was conducted at 14 UK hospitals. Patients with T2-T4aN0M0 muscle-invasive bladder cancer unsuitable for radical therapy received 36 Gy in 6 weekly fractions, randomized (1:1) to standard planning (SP) or adaptive planning (AP) using a minimization algorithm. For AP, a pretreatment cone beam computed tomography (CT) was used to select the POD from 3 plans (small, medium, and large). Follow-up included standard cystoscopic, radiologic, and clinical assessments. The primary endpoint was nongenitourinary Common Terminology Criteria for Adverse Events (CTCAE) grade ≥ 3 (≥G3) toxicity within 3 months of radiation therapy. A noncomparative single stage design aimed to exclude ≥30% toxicity rate in each planning group in patients who received ≥1 fraction of radiation therapy. Local control at 3-months (both groups combined) was a key secondary endpoint.

**Results:**

Between April 15, 2014, and August 10, 2016, 65 patients were enrolled (SP, *n* = 32; AP, *n* = 33). The median follow-up time was 38.8 months (interquartile range [IQR], 36.8-51.3). The median age was 85 years (IQR, 81-89); 68% of participants (44 of 65) were male; and 98% of participants had grade 3 urothelial cancer. In 63 evaluable participants, CTCAE ≥G3 nongenitourinary toxicity rates were 6% (2 of 33; 95% confidence interval [CI], 0.7%-20.2%) for the AP group and 13% (4 of 30; 95% CI, 3.8%-30.7%) for the SP group. Disease was present in 9/48 participants assessed at 3 months, giving a local control rate of 81.3% (95% CI, 67.4%-91.1%).

**Conclusions:**

POD adaptive radiation therapy was successfully implemented across multiple centers. Weekly ultrahypofractionated 36 Gy/6 fraction radiation therapy is safe and provides good local control rates in this older patient population.

## Introduction

Half of bladder cancer cases are diagnosed in patients aged over 75 years, many of whom are not fit for major surgery owing to their performance status and comorbidities or are unable to attend hospital for 4 to 7 weeks for daily radical radiation therapy.^1^, ^2^, ^3^, ^4^

This population presents a management dilemma, with unmet and potentially neglected clinical needs. A recent UK Royal College of Radiologist audit showed that just under 50% of patients with potentially curable T2 to T4 disease receive either no treatment or palliative radiation therapy only,^5^ similar to reports elsewhere.^6^^,^^7^ Despite their relatively poor performance status, many such patients have a life expectancy of several years and, if left untreated, may experience significant disease-related symptoms, such as hematuria, increased urinary frequency, dysuria, pelvic pain, urinary incontinence, and urinary obstruction.^8^

One option is to use ultrahypofractionated radiation therapy, which was shown to be equivalent to daily palliative radiation therapy treatment (35 Gy in 10 fractions) in a multicenter, randomized, phase 3 trial in muscle invasive bladder cancer (MRC BA09) that was conducted in the 1990s.^8^ However, the dose of 21 Gy in 3 fractions used in MRC BA09 and given over 1 week is relatively low, and the 3-month local cystoscopic control was modest (14 of 37; 38%). Several centers have tested an alternative ultrahypofractionated schedule of 6 Gy per fraction weekly, for a combined dose of 30 to 36 Gy over 5 to 6 weeks,^9^, ^10^, ^11^ which has a higher biological dose to the tumor than the BA09 schedules.^8^ Data on the 6 Gy per fraction schedule comes from retrospective reports and a single-center prospective study.^9^, ^10^, ^11^, ^12^

Bladder radiation therapy has traditionally used large margins between the clinical target volume (CTV) and planning target volume (PTV) to account for intrafraction variation in position and shape. The extra radiation caused by these large margins could add to toxicity, which presents a concern in this frail population. Modern image-guided adaptive radiation therapy protocols have been described to account for these changes, with a view to improving clinical outcomes. One option is to use a plan-of-the-day approach, where a best-fit plan from a pre-prepared library is used to more tightly conform to the target volume. This could be particularly important in the context of ultrafractionation, where each fraction represents over 15% of the total dose and missing a target or treating excessively could have a greater proportionate impact than those during conventional fractionation, where effects may be averaged out. This makes ultrafractionation an excellent context in which to test adaptive radiation therapy. Single-center feasibility results of this approach in this population, combined with ultrahypofractionated radiation therapy, have been reported.^12^

To test these approaches on a multicenter basis, we designed a non-comparative, randomized, phase 2 trial to assess the feasibility and clinic- and patient-reported outcomes of weekly ultrahypofractionated radiation therapy with and without adaptive radiation therapy in patients for whom conventional radical treatment for bladder cancer was unsuitable.

## Methods and Materials

### Study design

HYBRID (CRUK/12/055) is a non-comparative, multicenter randomized, phase 2 trial of ultrahypofractionated bladder radiation therapy with or without image guided adaptive planning in muscle-invasive bladder cancer from 14 National Health Service hospitals in the United Kingdom. The aims were to assess the feasibility and safety of delivering plan-of-the-day radiation therapy at multiple National Health Service sites and to assess the overall toxicity, patient-reported outcomes, and disease control associated with ultrahypofractionated radiotherapy.^13^

Eligible patients were aged at least 18 years, had histologically or cytologically confirmed bladder cancer staged T2 to T4a N0 M0, were unable to receive radical cystectomy or daily fractionated radiation therapy for any reason, had an expected survival of >6 months, and had a World Health Organization performance status of 0 to 3.

The key exclusion criteria were uncontrolled malignancy in the past 2 years, prior pelvic radiation therapy or major pelvic surgery, use of a urinary catheter, or any other contra-indication to radiation therapy.

Participants were recruited by their clinical care teams in the clinic and provided written informed consent before enrollment. The trial was registered ISRCTN18815596, approved by the London-Surrey Borders Research Ethics Committee (13/LO/1350), sponsored by the Institute of Cancer Research (ICR), and conducted in accordance with the principles of good clinical practice. The ICR Clinical Trials and Statistic Unit (ICR CTSU) coordinated the trial and carried out central statistical data monitoring and all analyses. The trial management group was overseen by independent data monitoring and trial steering committees. The full study protocol was published previously.^13^

### Randomization and masking

Treatment allocation was done centrally by ICR CTSU within 4 to 6 weeks before patients were due to start radiation therapy. Participants were assigned 1:1 between standard and adaptive planning using a minimization algorithm balanced for the radiation therapy treatment center and incorporating a random element. Treatment allocation was not masked.

### Procedures

Radiation therapy planning details and the quality assurance program are as described in Hafeez et al 2020^13^ In brief, all participants received 36 Gy in 6 fractions to the bladder. Participants in the standard planning group received radiation therapy using 1 plan throughout treatment. Three treatment plans (small, medium, and large) were generated for the adaptive planning (AP) group, with pre-RT cone beam (CB) computed tomography (CT) used to select the best fitting plan of the day for each fraction. A quality assurance program accredited individuals for plan selection. A single expert reviewer, (S.H.), blinded to outcomes, assessed concordance between online and offline plan selection retrospectively.

The CTV encompassed the visible tumor, whole bladder, and any area of extravesical spread. CTV to PTV expansion margins for SP and AP are given in Table E1. An example of derived PTVs is given in Fig. E6A. The margins for the adaptive planning are as derived from modeling work and validated in a subsequent single-center, phase 2 study.^12^^,^^14^^,^^15^

Comorbidity was assessed at baseline using the Charlson Comorbidity Index.^16^ A clinician assessment of toxicity was conducted weekly during treatment using the National Cancer Institute’s Common Terminology Criteria for Adverse Events (CTCAE) v4.0, with full blood count, urea, and electrolytes assessed at fractions 2, 4, and 6. CTCAE toxicity was subsequently assessed at 4 weeks and 3, 6, 12, and 24 months after the final radiation therapy fraction. Radiation Therapy Oncology Group (RTOG) toxicity was assessed by clinicians at 6, 12, and 24 months. A cystoscopic assessment of response was conducted at 3, 6, 12, and 24 months if possible, with urine cytology and a CT scan of the pelvis if the cystoscopic assessment was not possible. Participants were followed-up annually from 2 years for disease-related endpoints.

Patient-reported outcomes (PRO) were captured using the modified Inflammatory Bowel Disease Questionnaire (IBDQ; bowel function), the King’s Health Questionnaire (KHQ; urologic function), and the EuroQol 5-dimensions, 5-levels questionnaire (EQ5D; overall health status). Questionnaires were completed by participants on paper before radiation therapy, at fraction 6 and 3 and 6 months after completing radiation therapy.

### Outcomes

The primary endpoint was nongenitourinary (non-GU) CTCAE ≥G3 toxicity occurring within the first 3 months of radiation therapy completion. Secondary endpoints included the proportion of adaptive fractions delivered (ie, whether the small or large plan was selected; AP group only); appropriate identification of fractions requiring adaptive planning and selection of an appropriate plan; acute toxicity; late toxicity (up to 2 years); control rate of presenting symptoms; PROs assessed using the modified IBDQ, EQ5D, and KHQ; local disease control at 3 months; time to local disease progression; time to invasive local disease progression; and overall survival. Time to bladder cancer death was an exploratory endpoint.

Acute adverse events were categorized according to whether they emerged or worsened during treatment and their relationship to treatment. In this report, “adverse event” refers to an event that was not present at baseline or was reported at a higher grade than at baseline and “toxicity” refers to the subset of adverse events that were categorized as treatment related.

The categorization of relatedness of primary endpoint events to study treatment was reviewed by the independent data monitoring committee, which was blinded to the planning method.

### Statistical analysis

The study was designed to rule out an acute ≥G3 non-GU AP toxicity rate of 30%, assuming an expected rate of 10%.^12^ In each planning group, an A’Hern single-stage exact design (p0 = 0.70; p1 = 0.9; α = 0.05; β = 0·2), required at least 24/28 evaluable participants to be ≥G3 non-GU toxicity free for the hypofractionated plan of the day to be considered safe for radiation therapy. A 10% nonevaluable rate was accounted for in the target sample size of 62 patients.

Prospective power calculations were performed for a number of key secondary outcomes. With 62 participants, there was sufficient power to rule out a ≥G3 overall acute toxicity rate of hypofractionated radiation therapy of 40% or more (α = 0.05; β = 0.2) and a 3-month control rate (allowing for 25% nonevaluable patients) of less than 40% (α = 0.05; β = 0.13). It was estimated that approximately 50% of fractions in the AP group would be adapted.^12^ To assess the clinical utility of online correction, a threshold of 25% of all fractions or 1 fraction/patient requiring adaption was set. In the AP group, if true agreement between the online and offline protocols for plan selection was 85%, plan selection outcomes for 139 fractions would allow us to demonstrate agreement for >75% of fractions with 90% power (under a single-stage exact binomial approach).

There were no formal early stopping rules for efficacy or toxicity. However, an initial independent safety review took place when the 3-month data were available for 5 patients (who had received at least 3 fractions of radiation therapy) in each planning group.

For the primary endpoint, the evaluable population was all randomized patients who received at least 1 fraction of radiation therapy. This and other safety endpoints were analyzed according to the planning method received. Proportions are reported with 95% 2-sided exact binomial confidence intervals (CIs). For the primary and other key endpoints with prespecified threshold criteria, 90% 2-sided CIs are also provided (consistent with a 95% 1-sided design). Late toxicity is summarized by frequencies and proportions at each time point and overall assessments are shown by planning group and presented graphically. The time to the first instance of ≥G2 late toxicity was analyzed using competing risk methodology. Symptom control (graded the same, worse, or better than baseline) was assessed in patients with symptoms reported at baseline and is presented graphically for each planning group.

PRO data were analyzed in accordance with the relevant scoring manuals,^17^, ^18^, ^19^ with 3 months as the time point of primary interest. The modified IBDQ consists of 32 questions each, graded from 1 (worst possible symptom) to 7 (symptom absent or not changed since before radiation therapy). IBDQ total subscale scores were calculated by summing together all individual scores for patients with answers to all questions in that subscale.^17^ The KHQ is comprised of 3 parts with 21 items. For parts 1 and 2, items are scored between 0 (best) and 100 (worst) with a 4-point rating system, except for 1 item in part 1 (general health perceptions) that has a 5-point rating system.^18^ Part 3 is considered as a single item and is scored from 0 (best) to 27 (worst). The EQ5D consists of 5 domains, each graded from 1 (worst possible symptom) to 5 (symptom absent) except for 1 domain (pain) that is graded from 1 (worst possible symptom) to 4 (symptom absent).^19^ Changes in the mean scores were calculated from baseline to each posttreatment time point and were compared between randomized groups at 3 months using the analysis of covariance model, adjusting for baseline score. For the IBDQ and EQ5D, higher scores indicate better health; for the KHQ, lower scores reflect better health.

The local control rate at 3 months is presented for both planning groups combined. To consider the impact of early deaths and missed 3-month assessments, a sensitivity analysis was conducted and assumed: (1) bladder cancer–related deaths before the 3-month assessment were evidence of failure of local control; and (2) there was disease control at 3 months if the 3-month assessment was missing but the patient was reported as free of disease at a later time point with no intervening treatment.

Time-to-event endpoints, where the interest is in oncological outcomes of hypofractionation, are analyzed in the intention-to-treat population, summarized by Kaplan-Meier curves and presented for randomized groups combined. For overall survival, alive patients were censored on the date they were last seen. For other endpoints, patients with no events were censored on the date of last assessment of disease status (ie, date of last cystoscopy, biopsy, urine cytology, or CT scan). Patients who died before any follow-up were censored at the date of death.

Analyses were conducted using STATA version 15.0 based on a snapshot of data taken on June 10, 2019.

## Results

### Patient characteristics

**Fig. 1:**
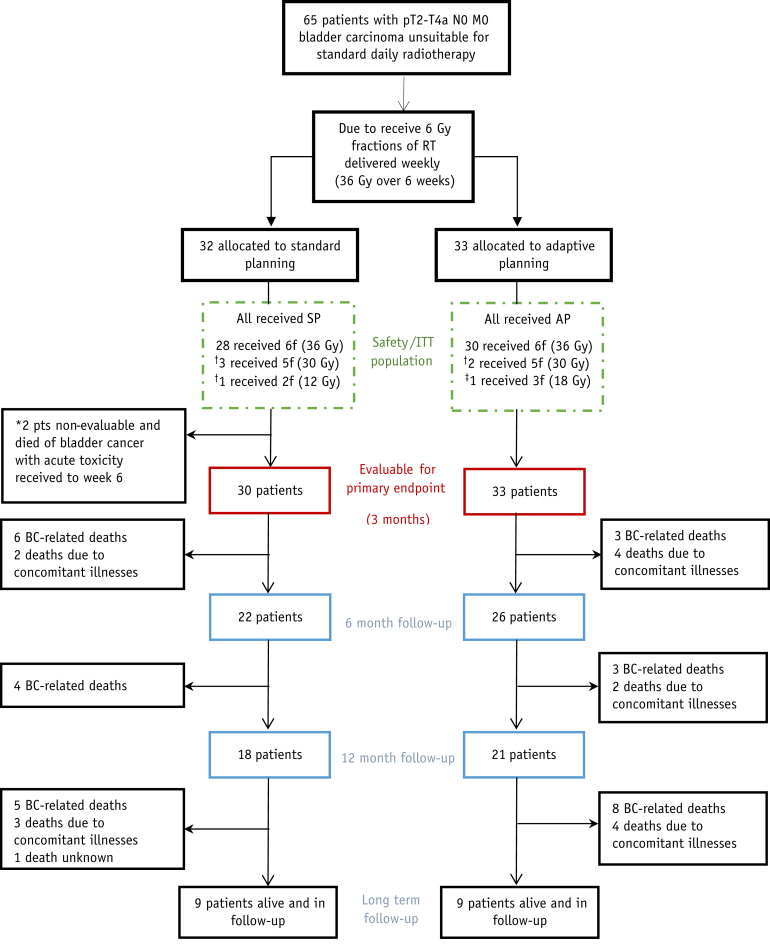
CONSORT diagram. ^∗^Classed as nonevaluable by Trial Steering Committee due to bladder cancer death before 3-month assessment or insufficient follow-up receipt. ^†^Stopped treatment early due to toxicity. ^‡^Stopped treatment early due to concomitant illness. *Abbreviation:* AP = adaptive planning; BC = bladder cancer; CONSORT = Consolidated Standards of Reporting Trials; f = fractions; ITT = intention to treat; RT = radiation therapy; SP = standard planning.

Between April 15, 2014, and August 10, 2016, 65 participants were recruited from 14 UK centers (Table E2). Of these, 32 patients were randomized to the SP group and 33 to the AP group (Fig. 1). All participants received radiation therapy given in accordance with their allocated planning method.

Patient characteristics are summarized in Table 1. The median age of participants was 85 years (interquartile range [IQR], 81-89), 68% (44 of 65) were male, and 98% had grade 3 urothelial carcinoma histology. A complete trans-urethral resection of the bladder tumor had been performed in 31%. The median age-adjusted Charlson comorbidity index score was 7, and scores ranged from 5 to 11 (IQR, 6-8).

**Table 1:** Baseline characteristics by allocated planning method

	Standard planning, n = 32	Adaptive planning, n = 33	Total, n = 65
Age, years	84.8 (80.7-88.9)	84.1 (80.4-87.1)	84.5 (80.6-88.6)
Months from histologic confirmation to randomization	1.7 (1.2-2.1)	1.4 (1.0-2.1)	1.6 (1.1-2.1)
Sex			
Male	24 (75%)	20 (61%)	44 (68%)
Female	8 (25%)	13 (39%)	21 (32%)
Extent of resection			
Biopsy	10 (31%)	10 (30%)	20 (31%)
Partial resection	10 (31%)	13 (39%)	23 (35%)
Full resection	11 (34%)	9 (27%)	20 (31%)
Unknown/missing	1 (3%)	1 (3%)	2 (3%)
Multiple tumors			
Yes	6 (19%)	7 (21%)	13 (20%)
No	26 (81%)	26 (79%)	52 (80%)
Histologic tumor type			
Urothelial	31 (97%)	32 (97%)	63 (97%)
Nonurothelial	1 (3%)	1 (3%)	2 (3%)
Grade			
G2	1 (3%)	0 (0%)	1 (2%)
G3	31 (97%)	33 (100%)	64 (98%)
CIS present			
Yes	9 (28%)	11 (33%)	20 (31%)
No	22 (69%)	22 (67%)	44 (68%)
Unknown/missing	1 (3%)	0 (0%)	1 (2%)
Clinical stage			
T2	24 (75%)	21 (64%)	45 (69%)
T3a	1 (3%)	5 (15%)	6 (9%)
T3b	5 (16%)	5 (15%)	10 (15%)
T4a	2 (6%)	2 (6%)	4 (6%)
NM stage			
N0/M0	32 (100%)	33 (100%)	65 (100%)
Age adjusted Charlson Comorbidity index score			
5	1 (3%)	0 (0%)	1 (2%)
6	14 (44%)	13 (39%)	27 (42%)
7	11 (34%)	5 (15%)	16 (25%)
8	4 (13%)	6 (18%)	10 (15%)
9	2 (6%)	8 (24%)	10 (15%)
10	0 (0%)	0 (0%)	0 (0%)
11	0 (0%)	1 (3%)	1 (2%)

*Abbreviation:* CIS = carcinoma in situ; IQR = interquartile range; NM stage = nodal/metastatic stage.

Data are shown as *n* (%) or median (IQR).

### Treatment delivery

Of the 65 patients, 58 (89%) completed 6 fractions of treatment, with the remaining 7 patients stopping early (Fig. 1).

In the AP group, 28 of 33 patients (85%) received at least 1 fraction using a plan other than the medium plan (95% CI, 68%-95%; Fig. E1), either using a small plan only throughout treatment (2 of 33 [6%]) or using 2 or more plans during treatment (2 plans, 22 of 33 [67%]; 3 plans, 4 of 33 [12%]). Overall, the number of fractions using a plan other than medium exceeded our target of 25%, with 76 of 193 fractions (39%; 95% CI, 32%-47%) delivered using a small plan (46 of 193; 24%) or a large plan (30 of 193; 16%).

Of 193 pretreatment CB CTs, 117 (60.6%) were available for central retrospective review, with a 78% (91 of 117) concordance rate between online plan selection and central review. In cases of discordance, the online plan selection was larger than the reviewer’s selection in 20 of 26 cases (77%; Table E3). The small plan was selected by the offline reviewer in 39 of 117 (33%) fractions, compared with 28 of 117 (24%) online selections.

### Acute toxicity

Toxicity rates were lower than the prespecified threshold in both groups. CTCAE ≥G3 non-GU toxicities were reported in 2 out of 33 participants (6%; 90% CI, 1.1%-17.9%; 95% CI, 0.7%-20.2%) in the AP group and 4 out of 30 (13%; 90% CI, 4.7%-28.0%; 95% CI, 3.8%-30.7%) in the SP group (Table 2). In each group, ≥G3 gastrointestinal toxicities were reported for 1 patient (included in non-GU toxicities).

**Table 2:** Acute ≥ grade 3 toxicities and adverse events

	Standard planning, n = 30		Adaptive planning, n = 33		Overall, n = 63	
	*n*	% (95% CI)	n	% (95% CI)	n	% (95% CI)
Toxicity						
Non-GU∗	4	13.3% (3.8-30.7)	2	6.1% (0.7-20.2)	6	9.5% (3.6-19.6)
GI†	1	3.3 % (0.1-17.2)	1	3.0% (0.1-15.8)	2	3.2% (0.4-11.0)
GU‡	5	17.2 % (5.8-35.8)	3	9.1% (1.9-24.3)	8	12.9% (5.7-23.9)
Any	7	23.3% (9.9-42.3)	5	15.2% (5.1-31.9)	12	19.0% (10.2-30.9)
Adverse event						
Non-GU	10	33.3% (17.3-52.8)	7	21.2% (9.0-38.9)	17	27.0% (16.6-39.7)
GI†	1	3.3% (0.1-17.2)	2	6.1% (0.7-20.2)	3	4.8% (1.0-13.3)
GU‡	8	27.6% (12.7-47.2)	3	9.1% (1.9-24.3)	11	17.7% (9.2-29.5)
Any	10	33.3% (17.3-52.8)	10	30.3% (15.6-48.7)	20	31.7% (20.6-44.7)

*Abbreviation:* GI = gastrointestinal; GU = genitourinary; SP = standard planning.

Adverse event refers to an event that was not present at baseline or was reported at a higher grade than at baseline, and toxicity refers to the subset of adverse events that were categorized as treatment related.

∗ This row shows the primary endpoint.

† GI is a subset of non-GU.

‡ One SP patient with GU had data that was not assessable. The patient was counted in the denominator.

The ≥G3 GU toxicities were more frequent than those that were non-GU, affecting 3 out of 33 (9%; 95% CI, 1.9%-24.3%) participants in the AP group and 5 out of 30 (17%; 95% CI, 5.8%-35.8%) in the SP group (Table 2).

**Fig. 2:**
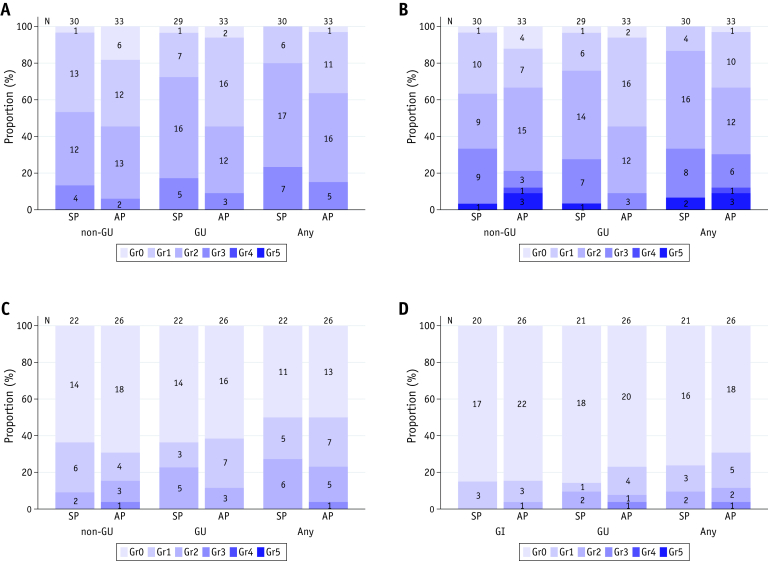
Stacked bar chart of the worst-grade acute toxicity, acute adverse event, late toxicity, and RTOG. Worst-grade (A) acute CTCAE toxicity, (B) acute CTCAE adverse event, (C) late CTCAE toxicity, and (D) RTOG. Adverse event refers to an event that was not present at baseline or was reported at a higher grade than at baseline, and toxicity refers to the subset of adverse events that were categorized as treatment related. *Abbreviation:* CTCAE = Common Terminology Criteria for Adverse Events; GI = gastrointestinal; GU = genitourinary; RTOG = Radiation Therapy Oncology Group.

The overall incidence of ≥G3 toxicity was lower than the predefined threshold rate of 40% (12 of 63; 19%; 90% CI, 11.4%-29.0%; 95% CI, 10.2%-30.9%). The distribution of overall, GU, and non-GU toxicities are shown in Fig. 2. No grade 4 or 5 toxicities were reported.

The equivalent data for all acute adverse events, regardless of relatedness to treatment, are shown in Table 2. Overall, 31.7% (20 out of 63) of patients had an acute ≥G3 acute adverse event.

### Late toxicity

Late toxicity is summarized in Fig. 2 and Fig. E2. Overall rates of late toxicity were low. In patients with at least 6 months of follow-up, 2 of 21 patients (9.5%) in the SP group and 3 of 26 (11.5%) in the AP group reported grade 2 or greater RTOG toxicity with a single episode of RTOG grade 3 toxicity reported in an AP patient (cystitis recorded at the 24-month follow-up). The time to first ≥G2 toxicity is shown in Fig. E3.

### Symptom control

The rate of control at 3 months of urinary symptoms is shown in Fig. E4 for each planning method separately. Hematuria, incontinence, and cystitis were improved in the majority of patients compared to baseline (hematuria, 12 of 16 [75%]; incontinence, 8 of 14 [57%]; cystitis, 11 of 19 [58%]). Frequency symptoms, though the same or better in most patients, only improved for the minority (nocturia, 8 of 39 [21%]; frequency/urgency, 12 of 36 [33%]).

### Patient-reported outcomes

**Fig. 3:**
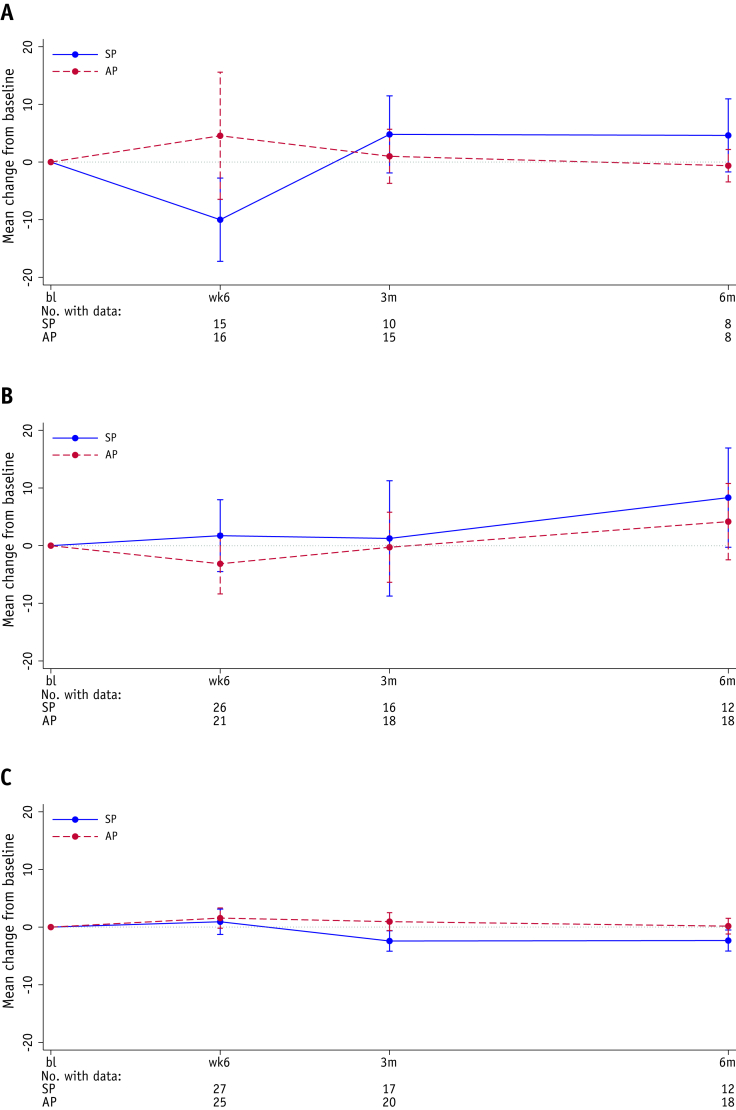
Mean change from baseline for the total IBDQ score, EQ5D health status and KHQ symptom severity score. Change from baseline in (A) IBDQ total score, (B) EQ5D health status score, and (C) KHQ Symptom severity measures score. Error bars represent 95% CIs. Negative numbers represent a decrease in quality of life and positive numbers represent an increase in quality of life for IBDQ and EQ5D. For KHQ, negative numbers represent an increase in quality of life and positive numbers represent a decrease in quality of life. *Abbreviation:* AP = adaptive planning, bl = baseline; CI = confidence interval; EQ5D = EuroQol 5-dimensions, 5-levels questionnaire; IBDQ = Inflammatory Bowel Disease Questionnaire; KHQ = King’s Health Questionnaire; SP = standard planning.

There was no statistically significant difference between the 2 planning groups for IBDQ bowel-related symptoms or for any other IBDQ symptoms, nor for the EQ5D health score or KHQ domain scores, at 3 months (Tables E4, E5 and E6; Fig. 3). Of note, the IBDQ demonstrated a worsening in bowel and systemic symptoms at Week 6 in the SP group which was not seen in the AP group (Table E4). This improved over the following 6 months and returned to baseline in both groups. The total IBDQ score, EQ5D health status, and KHQ symptom severity score over time are shown by patient in Fig. E5.

### Disease control and survival outcomes

At 3 months, 48 participants had their disease status assessed. Local disease was controlled in 39 out of 48 participants (81.3%; 90% CI, 69.6%-89.9%; 95% CI, 67.4%-91.1%). The rates of local control were 17 out of 23 (74%) in the SP group and 22 out of 25 (88%) in the AP group. A sensitivity analysis suggested at least 41 out of 61 patients (67.2%; 90% CI, 56.0%-77.1%; 95% CI, 54.0%-78.7%) had evidence of local control, thus, consistent with the main analysis, ruling out a 3-month control rate of less than 40%.

**Fig. 4:**
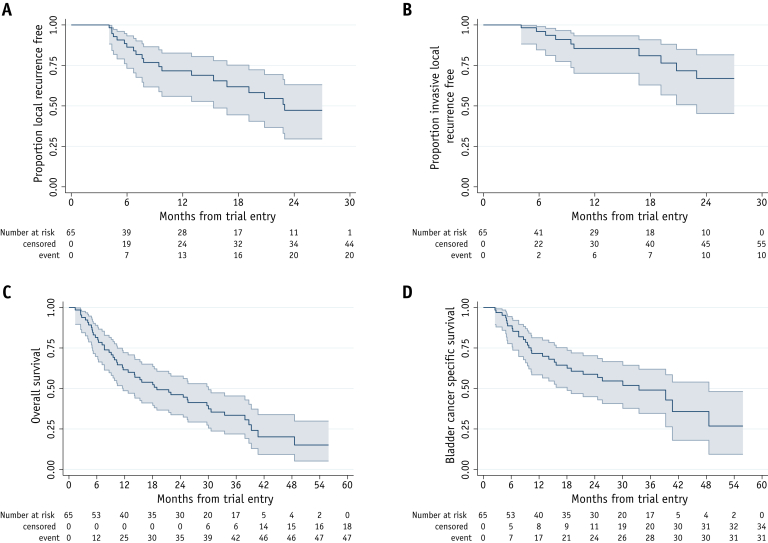
Kaplan-Meier plots of time to event. Time to (A) local recurrence, (B) local invasive recurrence, (C) overall survival, and (D) bladder cancer death. Number of events and number censored are presented as cumulative in the extended risk table. Shaded area represents 95% CI. *Abbreviation:* CI = confidence interval.

At a median of 38.8 months follow-up, 33 patients had reported 36 recurrences: 21 in the bladder (ie, local recurrence), 4 in the pelvic nodes, and a further 9 at distant sites (2 nodal and 7 at other distant sites). The proportion of patients free of local recurrence at 1 year was 71.7% (95% CI, 55.9%-82.6%) and the proportion free of invasive local recurrence was 85.5% (95% CI, 70.1%-93.3%; Fig. 4).

There have been 47 deaths, of which 31 are due to bladder cancer. The median survival time was 18.9 months, with 61.5% (95% CI, 48.6%-72.1%) alive at 1 year and 46.2% (95% CI, 33.8%-57.7%) alive at 2 years.

## Discussion

We set out to investigate whether the use of adaptive hypofractionated radiation therapy to a dose of 36 Gy in 6 fractions, in the context of a multicenter, prospective, randomized trial, could be an option for patients with advanced localized bladder cancer who were unable to receive standard radical treatment options. The study met its primary acute toxicity endpoint, ruling out excessive non-GU and overall toxicity and demonstrating that local control could be achieved in over 80% of participants at 3 months with this ultrahypofractionated weekly regimen. In ultrahypofractionated protocols, each fraction makes up a substantial proportion of the total treatment, so accuracy of delivery is important because it is difficult to compensate for even a single missed day. In this context we have, to the best of our knowledge, completed the first randomized trial of adaptive plan-of-the-day radiation therapy in bladder cancer. We have shown that a substantial portion of radiation therapy fractions may benefit from the use of a plan different to standard, which could have effects on toxicity and efficacy that may be particularly important in this elderly, frail population.

Treating this group of patients who, by virtue of age, performance status, or comorbidity are not fit for radical treatment but have potentially curable disease, is one of the large challenges facing clinicians treating muscle-invasive bladder cancer. This group of patients represents a substantial and understudied subset of patients. A recent UK study suggested that 47% of patients (representing 2519 patients per year) with T2 to T4 N0 bladder cancer are not receiving either radical radiation therapy or surgery.^20^

A remarkable feature of this trial was that it included a group of patients with a median age of 85 and significant comorbidity, as indicated by the Charlson Comorbidity index scores. The ability to complete this randomized study with good quality data, including data on local control and patient-reported outcomes, and with excellent adherence to allocated treatment shows that with an appropriate, flexible design it is possible to involve this patient population in research protocols, and they are willing to participate and be enrolled in trials.

Efficacy results from this phase 2 study are promising. A 3-month local control rate of over 80%; a 1-year invasive, local recurrence–free rate of 86%; and a median survival time of 18.9 months with over 40% of patients surviving 2 years posttreatment suggests that this schedule of 36 Gy in 6 fractions can be effective at controlling disease in patients with bladder cancer. Although on the face of it the survival figures are inferior to those reported in trials of chemo-radiotherapy,^21^ this treatment does still provide a reasonable chance of long-term survival and compares favorably with the 55% 1-year survival rate in patients receiving palliative treatments and the 32% survival rate with no treatment reported in the Royal College of Radiologist audit.^5^ In general, these results support the findings of the single-center, prospective, phase 2 study that was the pilot for this trial.^12^ In the pilot study, which included some patients with metastatic disease, there was local control in 92% of the 33 assessed patients (60% of all patients) with a 1-year survival rate of 63% and a 2-year survival rate of around 35%.^12^ Similar results have been reported for a number of retrospective studies^22^ and suggest the regimen of 36 Gy in 6 fractions may be superior to the hypofractionated schedule of 21 Gy in 3 fractions used in the MRC-BA09 trial, which reported a 38% local control rate in the small number of patients assessed.^8^

Although patients did experience a degree of acute toxicity, our trial met preset thresholds for acute tolerability, and late toxicity seemed uncommon. Thus, this study suggests that the regime of 36 Gy in 6 fractions weekly is a regime that can achieve local control in a significant proportion of patients and be tolerated even by an unfit population, making this a real treatment option for this patient population.

This study was also designed to develop preliminary clinical data on the value of an adaptive plan-of-the-day strategy. A significant body of evidence has accumulated showing that the changes in shape and position of the bladder through a treatment course can lead to a geographic miss despite the use of large CTV to PTV margins, which may contribute to increased toxicity in their own right. The advent of pre-treatment soft-tissue imaging has been used to develop a number of strategies to improve target coverage and reduce target margins. Foremost of these is the use of a plan of the day, where 1 of a set of predesigned plans of varying sizes is chosen. Previous work with plans of the day has resulted in target coverage higher than historical reports with reduction of the average PTV volume by 28% to 42%.^14^^,^^23^, ^24^, ^25^, ^26^

Our results here broadly support previous results, with 39% of treatments using either a small or large plan and most patients using either an adapted plan throughout or 2 or more of the 3 plans. This exceeds our minimum futility rate of 25% of treatments using an adapted plan and, as the medium plan used in this trial is smaller than a standard plan, 84% of treatments were treated with a plan smaller than normally used. This reflects reports from other studies of plan-of-the-day radiation therapy. Vestergaard et al^24^ reported roughly equal usage of small, medium, and large plans, resulting in a roughly 30% reduction in average PTV volume, whereas Foroudi et al^23^ used small or large plans for around 50% of fractions, resulting in a 29% reduction in the high-dose radiation volume.

It is encouraging that this study was deliverable in an environment where the technique was unfamiliar to most hospitals before their participation. As previously reported, all sites undertook a quality assurance program, including a training package on plan selection for which staff members needed to attain a preset level of concordance with an expert-defined selection to gain approval to select plans for the purposes of trial treatment.^27^^,^^28^ A central review shows this training was relatively effective, with 78% concordance, but in most discrepant cases the expert reviewer selected a smaller plan, evidencing the need for ongoing peer support and feedback in the implementation of this technique.

This study did have a number of limitations. Because it was a moderate-sized, noncomparative, phase 2 study, limited statements can be made about the benefits of adaptive radiation therapy compared with standard radiation therapy or of the 36 Gy/6 fraction schedule compared with other treatments. The data are also limited by early deaths and dropouts, meaning that not all patients could be assessed for toxicity, local control, and patient-reported outcomes. Despite these limitations, it is encouraging that the number of grade 3 to 4 non-GU and GU toxicity and adverse events are numerically lower in the adaptive arm. Additionally, fewer patients stopped radiation therapy early because of adverse events, and the higher immediate bowel-related quality of life is encouraging.

The trends in favor of improved outcomes for adaptive treatments should logically lead to a formal comparative study to confirm the degree of the clinical benefit from adaptive therapy, either in this patient group or in studies of patients receiving daily fractionated radiation therapy.

## Conclusions

Adaptive ultrahypofractionated radiation therapy is deliverable with modest toxicity in an elderly, unfit population of patients, while achieving local control for the majority. It represents a new option for care in this patient population.

## Supplementary Materials

Figs. E1-E6 and Table E1-E6
