# Supplementary material for: Clinical Outcomes of a Randomized Trial of Adaptive Plan-of-the-Day Treatment in Patients Receiving Ultra-hypofractionated Weekly Radiation Therapy for Bladder Cancer
Source: Int J Radiat Oncol Biol Phys. 2021 Jun 1;110(2):412–24. doi: 10.1016/j.ijrobp.2020.11.068 (PMC8114997; doi:10.1016/j.ijrobp.2020.11.068)
Supplement: Figs. E1-E6 and Table E1-E6 [file mmc1.docx]

**HYBRID supplementary materials**

**Table of contents**

**List of Tables**

Table E1. CTV to PTV expansion margins ………………………………………..…………………………….2

Table E2. Centre recruitment ………………………………………………………………………………………..2

Table E3. Plan concordance between actual plan delivered and independent reviewer selection …………………………………………………………………………………………..……………………………4

Table E4. Change from baseline in IBDQ symptoms per visit, per planning method and overall ……………………………………………………………………………………………………………………………7

Table E5. Change from baseline in EQ5D domains per visit, per planning method and overall ……………………………………………………………………………………………………………………………8

Table E6. Change from baseline in KHQ domains per visit, per planning method and overall ……………………………………………………………………………………………………………………………9

**List of Figures**

Figure E1. Plan concordance for use of adaptive planning per patient ………………………….3

Figure E2. Stacked bar chart of the worst grade non-GU, GU CTCAE toxicity and RTOG ..4

Figure E3. Time to first >G2 toxicity by planning method ……...………………………………………6

Figure E4. Symptom control at three months in patients reporting symptom at baseline ………………………………………………………………………………………………………………………………………7

Figure E5. Spider plot of change from baseline for the total IBDQ score, EQ5D health status and KHQ symptom severity score………………………………………………………………………11

Figure E6. Example of derived PTVs after margin expansion ………………………………………12

**Table E1 CTV to PTV expansion margins**

|  |  | CTV to Planning Target Volume Expansion (cm) | | | | |
| --- | --- | --- | --- | --- | --- | --- |
|  |  | Lateral | Anterior | Posterior | Superior | Inferior |
| SP | PTV | 1.5 | 1.5 | 1.5 | 1.5 | 1.5 |
| AP | PTV Small | 0.5 | 0.5 | 0.5 | 0.5 | 0.5 |
|  | PTV Medium | 0.5 | 1.5 | 1.0 | 1.5 | 0.5 |
|  | PTV Large | 0.8 | 2.0 | 1.2 | 2.5 | 0.8 |

SP = standard planning and AP = adaptive planning.

**Table E2 centre recruitment**

| **Centre** | **Principal Investigator** | **Total** |
| --- | --- | --- |
| Royal Marsden Hospital, Sutton | Robert Huddart (CI) | 12 |
| Clatterbridge Cancer Centre | Isabel Syndikus | 8 |
| St James’s University Hospital, Leeds | Ann Henry | 7 |
| Velindre Cancer Centre | John Staffurth | 7 |
| Royal Preston Hospital | Alison Birtle | 6 |
| Addenbrooke’s Hospital | Yvonne Rimmer | 4 |
| *Queen Elizabeth Hospital, King's Lynn | Gail Horan | 5 |
| *West Suffolk Hospital | Yvonne Rimmer | 1 |
| Ipswich Hospital | Ramachandran Venkitaraman | 4 |
| Royal Marsden Hospital, London | Vincent Khoo | 4 |
| UCLH | Anita Mitra | 3 |
| Guy's Hospital | Simon Hughes | 2 |
| Queen's Hospital Romford | Stephanie Gibbs | 1 |
| Norfolk and Norwich | Gaurav Kapur | 1 |

* Additional recruiting only centres for treatment at Addenbrooke's Hospital.

Figure E1.

Plans selected for each patient over course of treatment by local treating team and at retrospective central review.

**Table E3 Plan concordance between actual plan delivered and independent reviewer selection**

| Independent reviewer selection | Plan selected (Local investigator) | | | |
| --- | --- | --- | --- | --- |
|  | Small  N | Medium  N | Large  N | Total  N (%) |
| Small | 26 | 13 | 0 | 39 (33) |
| Medium | 2 | 55 | 7 | 64 (55) |
| Large | 0 | 4 | 10 | 14 (12) |
| Total (%) | 28 (24) | 72 (62) | 17 (15) | 117 (100) |

**Late toxicity**

**Figure E2 Stacked bar chart of the worst grade non-GU, GU CTCAE toxicity and RTOG**

A. Worst grade non-GU CTCAE toxicity, B. Worst grade GU CTCAE toxicity and C. Worst grade RTOG. SP = standard planning, AP = adaptive planning, Gr = grade.

**Figure E3 Time to first >G2 toxicity by planning method**

SP = standard planning, AP = adaptive planning. Number of events and number at risk are presented cumulative in the risk table. Competing risk analysis has been carried out with local recurrence or death as competing events and patients with no event have been censored at their last toxicity assessment.

**Symptom control**

**Figure E4 Symptom control at three months in patients reporting symptom at baseline**

**(Renal and urinary symptom reported in 20% or more of patients)**

SP = standard planning, AP = adaptive planning. Baseline symptoms are those recorded at fraction one, prior to starting radiotherapy. If an assessment at fraction one was not available, the baseline/screening visit was used.

**PRO**

**Table E4 Change from baseline in IBDQ symptoms per visit, per planning method and overall**

|  |  | Standard Planning | | | | Adaptive Planning | | | Overall | | | p-value* |
| --- | --- | --- | --- | --- | --- | --- | --- | --- | --- | --- | --- | --- |
|  | Visit | N | Mean | 95% CI | N | | Mean | 95% CI | N | Mean | 95% CI |  |
| IBDQ total Score | Week 6 | 15 | -10·0 | -17·2, -2·8 | 16 | | 4·6 | -6·5, 15·6 | 31 | -2·5 | -9·4, 4·4 |  |
|  | 3 month | 10 | 4·8 | -1·9, 11·5 | 15 | | 1·0 | -3·7, 5·7 | 25 | 2·5 | -1·3, 6·4 | 0·781 |
|  | 6 month | 8 | 4·6 | -1·7, 11·0 | 8 | | -0·6 | -3·4, 2·2 | 16 | 2·0 | -1·5, 5·5 |  |
| Bowel symptoms | Week 6 | 23 | -8·0 | -10·2, -5·8 | 21 | | -1·6 | -5·5, 2·2 | 44 | -5·0 | -7·2, -2·7 |  |
|  | 3 month | 11 | -0·7 | -1·8, 0·4 | 15 | | -1·2 | -2·3, -0·1 | 26 | -1·0 | -1·8, -0·2 | 0·738 |
|  | 6 month | 9 | 0·4 | -1·2, 2·1 | 12 | | -0·8 | -1·8, 0·1 | 21 | -0·3 | -1·2, 0·6 |  |
| Systemic symptoms | Week 6 | 26 | -5·2 | -7·4, -2·9 | 21 | | 0·0 | -1·8, 1·9 | 47 | -2·8 | -4·4, -1·2 |  |
|  | 3 month | 15 | -1·8 | -5·5, 1·9 | 18 | | 0·6 | -1·4, 2·5 | 33 | -0·5 | -2·5, 1·5 | 0·270 |
|  | 6 month | 11 | 2·5 | -0·8, 5·9 | 12 | | 2·5 | -1·3, 6·3 | 23 | 2·5 | 0·1, 5·0 |  |
| Emotional symptoms | Week 6 | 20 | -3.5 | -7.0, -0.0 | 21 | | 0.5 | -4.1, 5.1 | 41 | -1.5 | -4.4, 1.5 |  |
|  | 3 month | 12 | 6.0 | 1.9, 10.1 | 16 | | 1.6 | -0.1, 3.4 | 28 | 3.5 | 1.4, 5.6 | 0·572 |
|  | 6 month | 8 | 2.8 | -0.2, 5.7 | 11 | | 1.2 | -0.9, 3.2 | 19 | 1.8 | 0.1, 3.5 |  |
| Social symptoms | Week 6 | 18 | -1·0 | -2·2, 0·2 | 19 | | 0·5 | -0·8, 1·9 | 37 | -0·2 | -1·1, 0·7 |  |
|  | 3 month | 13 | 1·0 | -0·2, 2·2 | 17 | | -0·1 | -0·9, 0·8 | 30 | 0·4 | -0·3, 1·1 | 0·216 |
|  | 6 month | 10 | 0·4 | -0·5, 1·3 | 10 | | -0·2 | -0·4, 0·0 | 20 | 0·1 | -0·3, 0·5 |  |

* P-value: using the ANCOVA model and adjusting for baseline score

Negative numbers represent a decrease in quality of life and positive numbers an increase in quality of life from baseline

**Table E5 Change from baseline in EQ5D domains per visit, per planning method and overall**

|  |  | Standard Planning | | | | Adaptive Planning | | | Overall | | | p-value* |
| --- | --- | --- | --- | --- | --- | --- | --- | --- | --- | --- | --- | --- |
|  | Visit | N | Mean | 95% CI | N | | Mean | 95% CI | N | Mean | 95% CI |  |
| Mobility | Week 6 | 25 | 0·0 | -0·3, 0·3 | 24 | | 0·3 | 0·1, 0·6 | 49 | 0·2 | -0·0, 0·4 |  |
|  | 3 month | 16 | 0·1 | -0·4, 0·6 | 18 | | 0·2 | -0·1, 0·5 | 34 | 0·1 | -0·1, 0·4 | 0·632 |
|  | 6 month | 12 | 0·1 | -0·4, 0·6 | 17 | | 0·3 | -0·1, 0·7 | 29 | 0·2 | -0·1, 0·5 |  |
| Self-care | Week 6 | 24 | 0·1 | -0·1, 0·2 | 23 | | 0·1 | -0·2, 0·5 | 47 | 0·1 | -0·1, 0·3 |  |
|  | 3 month | 14 | -0·2 | -0·5, 0·0 | 18 | | 0·2 | -0·3, 0·7 | 32 | 0·0 | -0·3, 0·3 | 0·637 |
|  | 6 month | 10 | 0·1 | -0·2, 0·4 | 16 | | 0·1 | -0·4, 0·6 | 26 | 0·1 | -0·2, 0·4 |  |
| Usual activities | Week 6 | 24 | -0·3 | -0·6, 0·1 | 24 | | 0·2 | -0·2, 0·6 | 48 | -0·0 | -0·3, 0·2 |  |
|  | 3 month | 15 | -0·1 | -0·6, 0·3 | 18 | | 0·0 | -0·5, 0·5 | 33 | -0·1 | -0·4, 0·3 | 0·546 |
|  | 6 month | 10 | 0·1 | -0·6, 0·8 | 17 | | 0·2 | -0·3, 0·8 | 27 | 0·2 | -0·2, 0·6 |  |
| Pain/discomfort | Week 6 | 25 | 0·0 | -0·2, 0·3 | 24 | | -02 | -0·5, 0·2 | 49 | -0·1 | -0·3, 0·2 |  |
|  | 3 month | 16 | -0·1 | -0·4, 0·3 | 18 | | 01 | -0·3, 0·4 | 34 | 0·0 | -0·3, 0·3 | 0·801 |
|  | 6 month | 11 | 0·0 | -0·2, 0·2 | 16 | | -0·1 | -0·6, 0·3 | 27 | -0·1 | -0·4, 0·2 |  |
| Anxiety/depression | Week 6 | 23 | 0·0 | -0·4, 0·4 | 24 | | 0·0 | -0·2, 0·3 | 47 | 0·0 | -0·2, 0·2 |  |
|  | 3 month | 15 | 0·2 | -0·1, 0·5 | 18 | | -0·1 | -0·3, 0·2 | 33 | 0·1 | -0·1, 0·3 | 0·750 |
|  | 6 month | 10 | 0·2 | -0·4, 0·8 | 17 | | 0·0 | -0·3, 0·3 | 27 | 0·1 | -0·2, 0·4 |  |
| Health status | Week 6 | 26 | 1·7 | -4·5, 8·0 | 21 | | -3·1 | -8·4, 2·1 | 47 | -0·4 | -4·6, 3·7 |  |
|  | 3 month | 16 | 1·3 | -8·8, 11·3 | 18 | | -0·3 | -6·4, 5·8 | 34 | 0·4 | -5·2, 6·1 | 0·618 |
|  | 6 month | 12 | 8·3 | -0·3, 16·9 | 18 | | 4·2 | -2·5, 10·8 | 30 | 5·8 | 0·6, 11·0 |  |

* P-value: using the ANCOVA model and adjusting for baseline score

Negative numbers represent a decrease in quality of life and positive numbers an increase in quality of life from baseline

**Table E6 Change from baseline in KHQ domains per visit, per planning method and overall**

|  |  | Standard Planning | | | Adaptive Planning | | | Overall | | | p-value* |
| --- | --- | --- | --- | --- | --- | --- | --- | --- | --- | --- | --- |
|  |  | N | Mean | 95% CI | N | Mean | 95% CI | N | Mean | 95% CI |  |
| General health | Week 6 | 27 | 4·6 | -3.0, 12.2 | 23 | 0.0 | -5.8, 5.8 | 50 | 2.5 | -2.4, 7.4 |  |
| perceptions | 3 month | 17 | 7.4 | -1.8, 16.5 | 18 | -2.8 | -9.3, 3.8 | 35 | 2.1 | -3.5, 7.8 | 0.149 |
|  | 6 month | 12 | 0.0 | -7.2, 7.2 | 16 | -3.1 | -14.3, 8.1 | 28 | -1.8 | -8.8, 5.2 |  |
| Incontinence impact | Week 6 | 27 | 6.2 | -2.1, 14.5 | 23 | 7.2 | -1.2, 15.7 | 50 | 6.7 | 0.8, 12.5 |  |
|  | 3 month | 17 | -7.8 | -20.7, 5.1 | 18 | 3.7 | -6.1, 13.5 | 35 | -1.9 | -10.0, 6.2 | 0.910 |
|  | 6 month | 12 | -13.9 | -29.7, 1.9 | 16 | 6.3 | -5.2, 17.7 | 28 | -2.4 | -12.1, 7.4 |  |
| Role limitations | Week 6 | 25 | 12.7 | 2.3, 23.1 | 24 | 6.3 | -0.3, 12.8 | 49 | 9.5 | 3.3, 15.7 |  |
|  | 3 month | 16 | 2.1 | -6.2, 10.3 | 20 | 9.2 | 1.1, 17.2 | 36 | 6.0 | 0.2, 11.8 | 0.471 |
|  | 6 month | 11 | -6.1 | -16.7, 4.6 | 15 | 6.7 | -3.6, 17.0 | 26 | 1.3 | -6.3, 8.9 |  |
| Physical limitations | Week 6 | 24 | 19.4 | 7.7, 31.2 | 24 | 4.9 | -3.3, 13.0 | 48 | 12.2 | 4.9, 19.4 |  |
|  | 3 month | 16 | 5.2 | -6.2, 16.6 | 20 | 10.8 | 1.7, 20.0 | 36 | 8.3 | 1.2, 15.5 | 0.878 |
|  | 6 month | 11 | 4.5 | -9.8, 18.9 | 15 | 8.9 | -1.1, 18.9 | 26 | 7.1 | -1.2, 15.3 |  |
| Social limitations | Week 6 | 23 | 21.5 | 11.7, 31.3 | 23 | 2.4 | -4.5, 9.3 | 46 | 12.0 | 5.6, 18.3 |  |
|  | 3 month | 16 | 11.1 | 3.1, 19.1 | 19 | 10.5 | 2.0, 19.1 | 35 | 10.8 | 5.0, 16.6 | 0.926 |
|  | 6 month | 11 | -1.0 | -12.4, 10.4 | 14 | 2.4 | -4.6, 9.3 | 25 | 0.9 | -5.3, 7.1 |  |
| Personal | Week 6 | 20 | 7.5 | 2.8, 12.2 | 19 | 6.1 | 0.1, 12.1 | 39 | 6.8 | 3.1, 10.6 |  |
| relationships | 3 month | 12 | 9.7 | -4.0, 23.4 | 16 | 6.2 | 0.7, 11.8 | 28 | 7.7 | 1.2, 14.3 | 0.674 |
|  | 6 month | 9 | 3.7 | -0.3, 7.7 | 12 | 8.3 | -3.1, 19.8 | 21 | 6.3 | -0.3, 13.0 |  |
| Emotions | Week 6 | 26 | 9.4 | -1.2, 20.0 | 21 | 2.6 | -1.3, 6.6 | 47 | 6.4 | 0.2, 12.5 |  |
|  | 3 month | 16 | 2.8 | -8.0, 13.5 | 18 | -0.0 | -11.0, 11.0 | 34 | 1.3 | -6.3, 8.9 | 0.068 |
|  | 6 month | 12 | -10.2 | -22.8, 2.4 | 11 | 4.0 | -2.6, 10.7 | 23 | -3.4 | -11.0, 4.2 |  |
| Sleep and energy | Week 6 | 26 | 15.4 | 6.4, 24.4 | 23 | 5.8 | -0.3, 11.9 | 49 | 10.9 | 5.3, 16.5 |  |
|  | 3 month | 16 | 1.0 | -10.6, 12.6 | 19 | -4.4 | -9.9, 1.1 | 35 | -1.9 | -7.9, 4.1 | 0.196 |
|  | 6 month | 12 | -5.6 | -20.8, 9.7 | 12 | -1.4 | -10.0, 7.2 | 24 | -3.5 | -12.0, 5.1 |  |
| Severity measures | Week 6 | 26 | 5.9 | -0.2, 11.9 | 22 | 1.5 | -3.6, 6.6 | 48 | 3.9 | -0.1, 7.9 |  |
|  | 3 month | 16 | -1.2 | -8.3, 5.9 | 19 | 3.5 | -4.8, 11.9 | 35 | 1.3 | -4.2, 6.9 | 0.874 |
|  | 6 month | 12 | -1.4 | -7.7, 4.9 | 12 | 4.2 | -6.6, 14.9 | 24 | 1.4 | -4.8, 7.5 |  |
| Symptom severity | Week 6 | 27 | 0.9 | -1.3, 3.1 | 25 | 1.6 | -0.2, 3.3 | 52 | 1.2 | -0.2, 2.6 |  |
| measures | 3 month | 17 | -2.4 | -4.2, -0.6 | 20 | 0.9 | -0.6, 2.5 | 37 | -0.6 | -1.8, 0.6 | 0.151 |
|  | 6 month | 12 | -2.3 | -4.2, -0.5 | 18 | 0.2 | -1.2, 1.5 | 30 | -0.8 | -2.0, 0.3 |  |

* P-value: using the ANCOVA model and adjusting for baseline score

Negative numbers represent an increase in quality of life and positive numbers a decrease in quality of life from baseline

**Figure E5 Spider plot of change from baseline for the total IBDQ score, EQ5D health status and KHQ symptom severity score**

Change from baseline in (A) IBDQ total score (B) EQ5D health status score and (C) KHQ Symptom severity measures score SP = standard planning, AP = adaptive planning, bl = baseline. Negative numbers represent a decrease in quality of life and positive numbers an increase in quality of life for IBDQ and EQ5D. For KHQ negative numbers represent an increase in quality of life and positive numbers a decrease in quality of life.

**Figure E6 Example of derived PTVs after margin expansion**


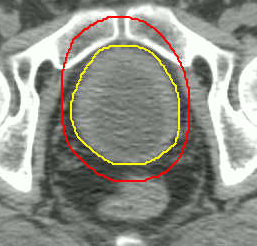

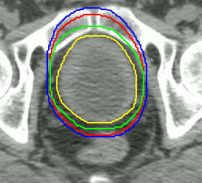

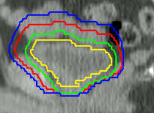

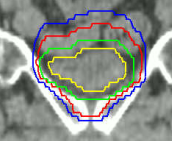


A

B

C

Bladder CTV is drawn in yellow line. The derived PTV volumes are shown in a. axial b. sagittal and c coronal planes

Small PTV green lines; Medium PTV red lines; Large PTV blue lines
